# Cerebral Microvascular Accumulation of Tau Oligomers in Alzheimer’s Disease and Related Tauopathies

**DOI:** 10.14336/AD.2017.0112

**Published:** 2017-05-02

**Authors:** Diana L Castillo-Carranza, Ashley N Nilson, Candice E Van Skike, Jordan B Jahrling, Kishan Patel, Prajesh Garach, Julia E Gerson, Urmi Sengupta, Jose Abisambra, Peter Nelson, Juan Troncoso, Zoltan Ungvari, Veronica Galvan, Rakez Kayed

**Affiliations:** ^1^Mitchell Center for Neurodegenerative Diseases, University of Texas Medical Branch, Galveston, TX 77555, USA; ^2^Departments of Neurology, Neuroscience and Cell Biology, University of Texas Medical Branch, Galveston, TX 77555, USA; ^3^Sealy Center for Vaccine Development, University of Texas Medical Branch, Galveston, TX 77555, USA; ^4^Department of Cellular and Integrative Physiology and The Barshop Institute for Longevity and Aging Studies, University of Texas Health Science Center at San Antonio, TX 78245, USA; ^5^Sanders-Brown Center on Aging and Department of Physiology, University of Kentucky, Lexington, KY 40536, USA; ^6^Division of Neuropathology and Sanders-Brown Center on Aging, University of Kentucky, Lexington, KY 40536, USA; ^7^Clinical and Neuropathology Core, Department of Medicine, Johns Hopkins University, Baltimore, MD 21287, USA; ^8^Department of Geriatric Medicine and Reynolds Oklahoma Center on Aging, University of Oklahoma Health Sciences Center, Oklahoma, OK 73104, USA

**Keywords:** tau, oligomers, tauopathies, cerebrovascular dysfunction, brain vascular dysfunction, cerebrovasculature, Alzheimer’s disease

## Abstract

The importance of vascular contributions to cognitive impairment and dementia (VCID) associated with Alzheimer’s disease (AD) and related neurodegenerative diseases is increasingly recognized, however, the underlying mechanisms remain obscure. There is growing evidence that in addition to Aβ deposition, accumulation of hyperphosphorylated oligomeric tau contributes significantly to AD etiology. Tau oligomers are toxic and it has been suggested that they propagate in a “prion-like” fashion, inducing endogenous tau misfolding in cells. Their role in VCID, however, is not yet understood. The present study was designed to determine the severity of vascular deposition of oligomeric tau in the brain in patients with AD and related tauopathies, including dementia with Lewy bodies (DLB) and progressive supranuclear palsy (PSP). Further, we examined a potential link between vascular deposition of fibrillar Aβ and that of tau oligomers in the Tg2576 mouse model. We found that tau oligomers accumulate in cerebral microvasculature of human patients with AD and PSP, in association with vascular endothelial and smooth muscle cells. Cerebrovascular deposition of tau oligomers was also found in DLB patients. We also show that tau oligomers accumulate in cerebral microvasculature of Tg2576 mice, partially in association with cerebrovascular Aβ deposits. Thus, our findings add to the growing evidence for multifaceted microvascular involvement in the pathogenesis of AD and other neurodegenerative diseases. Accumulation of tau oligomers may represent a potential novel mechanism by which functional and structural integrity of the cerebral microvessels is compromised.

There is growing evidence that a spectrum of vascular and microvascular pathologies (including neurovascular uncoupling, endothelial dysfunction, blood brain barrier disruption, cerebral microhemorrhages etc) contribute to initiation and/or progression of neurodegenerative diseases, including Alzheimer’s disease (AD) [1-4]. To recognize this paradigm, shift the phrase “vascular contributions to cognitive impairment and dementia (VCID)” was introduced [5-7]. The VCID concept provides mechanistic explanation to the well-established role of known vascular risk factors in exacerbation of AD. Results from recent research suggest that VCID are manifested early during development of AD [8] and are amenable for therapeutic intervention to dementia.

The cellular and molecular mechanisms underlying VCID are not well understood. There are many studies extant that attribute cerebrovascular abnormalities in AD to the deposition of fibrillar Aβ in the wall of cerebral vessels [9-12], a pathology referred to as cerebral amyloid angiopathy (CAA). However, there is increasing evidence that other molecular mechanisms also play an equally important role. Importantly, in addition to extracellular accumulation of Aβ the intracellular accumulation of the microtubule-associated protein tau is believed to contribute to the pathogenesis of neurodegeneration [13-15]. Under normal conditions tau binds to microtubules and assists with their formation and stabilization. In AD and other related neurodegenerative disorders (collectively called tauopathies) tau is hyperphosphorylated and misfolded, and accumulates into insoluble intracellular aggregates known as neurofibrillary tangles (NFT) [16, 17] which likely interfere with numerous cellular functions. In addition, recent research suggest that multimers comprised of two or more tau molecules, called tau oligomers [18] are a distinct toxic tau species and have the ability to translocate between neurons [19-21]. Tau oligomers thus exhibit “prion-like” propagation mechanisms and are capable of spreading in the brain by inducing endogenous tau phosphorylation and misfolding [22]. In addition to AD, tau oligomers have been identified in other tauopathies including progressive supranuclear palsy (PSP) [23], Parkinson’s disease (PD), dementia with Lewy bodies (DLB) [24] and in subjects with Huntington’s disease (HD) [25] suggesting a common pathogenic role. Despite these advances, the role of oligomeric tau in cerebromicrovascular abnormalities associated with neurodegenerative diseases remains obscure.

The goal of the present study was to determine vascular deposition of oligomeric tau in the brain in patients with AD and other types of tauopathies, including DLB and PSP. Further, we examined a potential link between CAA and vascular deposition of tau oligomers in the Tg2576 mouse model.

## MATERIALS AND METHODS

### Human samples

Postmortem brain samples from AD, DLB, PSP, and age-matched control subjects, were obtained from Oregon Health and Science University, the Institute for Brain Aging and Dementia (University of California-Irvine, Irvine, California, USA) and the Brain Resource Center at Johns Hopkins.

### Animals

All studies were performed under approval of the UTMB Institutional Animal Care and Use Committee [Animal Welfare Assurance Number D16-00202 (A3314-01)]. Tg2576 mice were bred at UTMB free of enrichment. Mice were housed at the UTMB animal care facility and maintained according to U.S. Department of Agriculture standards (12 h light/dark cycle with food and water available *ad libitum*). Tg2576 mice overexpress amyloid precursor protein (APP) carrying the KM670/671NL (‘Swedish’) mutation and develop early amyloid plaques and abundant vascular amyloid deposition [26].. The brains of Tg2576 mice were examined at 3 and 23 months of age.

### Immunohistochemistry

Immunohistochemistry was performed on paraffin-embedded sections. Briefly, sections were deparaffinized using xylene and ethyl alcohol and subsequently rehydrated and washed three times in 1X phosphate buffered saline (PBS) for 5 minutes each. Non-specific antigens were blocked using normal goat serum at room temperature for 1 hour. Sections were incubated overnight with primary rabbit polyclonal anti-tau oligomer antibody (T22, 1:200). Sections were washed three times in PBS for 10 min and incubated with goat anti-rabbit IgG for 1 hour at room temperature. (Vectastin ABC kit; Vector Laboratories, Burlingame, CA). A 3, 3′-diaminobenzidine peroxidase substrate kit (Vector Laboratories) was used to visualize tau oligomer immunoreactivity. Hematoxylin was used to counterstain nuclei according to the manufacturer’s instructions. Bright field images were acquired using a Nikon Eclipse 800 microscope equipped with a Nikon DXM1200 color CCD camera (Nikon Instruments, Inc., Melville, NY).

### Immunofluorescence

Fluorescent immunohistochemistry was performed on paraffin-embedded sections. Briefly, paraffin sections were deparaffinized and rehydrated. Sections were blocked with goat serum for 1 hour and incubated overnight at 4°C with rabbit anti-tau oligomer antibody T22 (1:200). The next day, sections were washed with PBS and incubated with goat anti-rabbit IgG Alexa-568 (1:350, Invitrogen) for 1 hour. The sections were then washed three times with PBS, followed by blocking in 5% goat serum for 1 hour, then by overnight incubation with a smooth muscle actin antibody [SMA, 1:250 (0.8 μg/ml), Abcam], Lewy body antibody [LB509, 1:300 (3 μg/ml), Abcam], von Willebrand Factor antibody [vWF, 1:250 (4 μg/ml), Chemicon], Aβ antibody [6E10, 1:300 (3.33 μg/ml), Biolegend], or total tau antibody [Tau 5, 1:300 (0.16 μg/ml), Biolegend]. The next day, the sections were washed three times in PBS, followed by 1 hour incubation with goat anti-rabbit IgG or anti-mouse Alexa Fluor 488 (1:350; Invitrogen) depending on the species the primary antibody was raised in. The sections were then washed three times in PBS, stained with DAPI (Vector Laboratories) and mounted in Vectashield mounting media. The sections were examined using an epifluorescence microscope (Nikon Eclipse 800) equipped with a CoolSnap-FX monochrome CCD camera (Photometrics, Tucson, AZ, USA) using standard Nikon fluorescein isothiocyanate (FITC), Texas Red, and DAPI filters set for Alexa Fluor 488, Alexa Fluor 568 and DAPI respectively. Imaging and analysis were performed using Metavue V7.1 software (Molecular Devices), Adobe Photoshop and FIJI/Image J.

**Figure 1.**
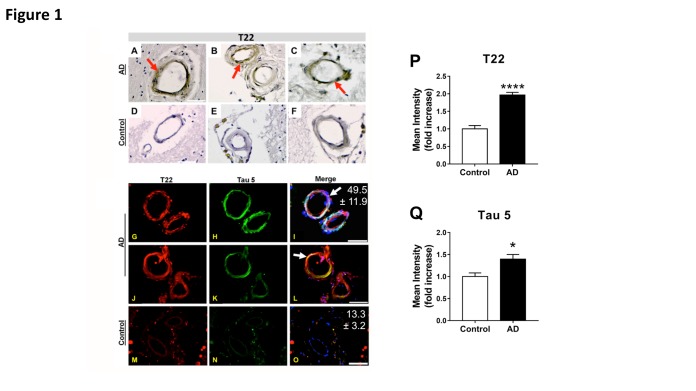
Deposition of tau oligomers in cerebrovasculature of human Alzheimer’s Disease (AD) brains **(A-F)** Representative images of immunohistochemistry studies using an oligomeric tau-specific antibody (T22) in cortical sections from AD **(A-C)** and age-matched controls **(D-F)**. **(G-O)** Representative images of oligomeric tau (T22, red), and total tau (tau 5, green) cerebrovascular immunoreactivity in cortical sections of AD **(G-L)** and age-matched control **(M-O)** brains. Quantitative analyses of mean fluorescence intensity show increased tau oligomer- (T22, **P**) and total tau-specific (Tau5, **Q**) immunoreactivity in vasculature of AD brains compared to age-matched controls [****, t (28) = 8.12, *p* < 0.0001, and *, t(17) = 2.39, *p* = 0.029, for T22 and tau5 immunoreactivity respectively]. Our tau oligomer antibody T22 [18, 41] has been validated by immunoblot, ELISA, coimmunoprecipitation as well as rodent and human tissue staining, is produced endotoxin-free, and is commercially available (Millipore ABN454). For all studies, n=3 brains/group; 10-15 sections from each sample were analyzed for tau oligomers. All AD samples were tested and were positive for tau oligomers. Merged images are shown with DAPI (blue). In all panels, arrows indicate tau inclusions. Mean percent colocalization ± SEM of T22 with Tau 5 is reported in the figure. Scale bar 50 µm.

### Confocal imaging

Images were collected using a Zeiss LSM-510 Meta confocal microscope with a 63 × 1.20 numerical aperture water immersion objective (UTMB Optical Microscopy Core). The images were obtained using three different excitation lines (364, 488, and 543) by sequential acquisition. After excitation with 364-, 488- and 543-lasers, line emissions were collected with 385-470-nm, 505-530-nm, and 560-615-nm filters, respectively. All images were collected using 8-frame-Kallman-averaging with a pixel time of 2.51μs and a pixel size of 160 nm. Image processing and analysis were performed with Metamorph 7.2, LSM Image browser, and FIJI/Image J.

### Image analysis and statistics

To obtain mean intensity measurements, mean gray values of immunofluorescent images were determined using the threshold technique in Image J. Briefly, original 8-bit grayscale images were uploaded, the threshold was adjusted to match the immunofluorescence contained in the image, single vascular elements were isolated with the selection tool, and the mean gray value of the thresholded area was measured and recorded. When applicable, Mander’s colocalization coefficient was computed using the “coloc 2” plugin in the FIJI version of Image J. All differences between two group means were assessed with t-tests. When comparing 3 group means, a one-way ANOVA was used, followed by Tukey’s posthoc.

**Figure 2.**
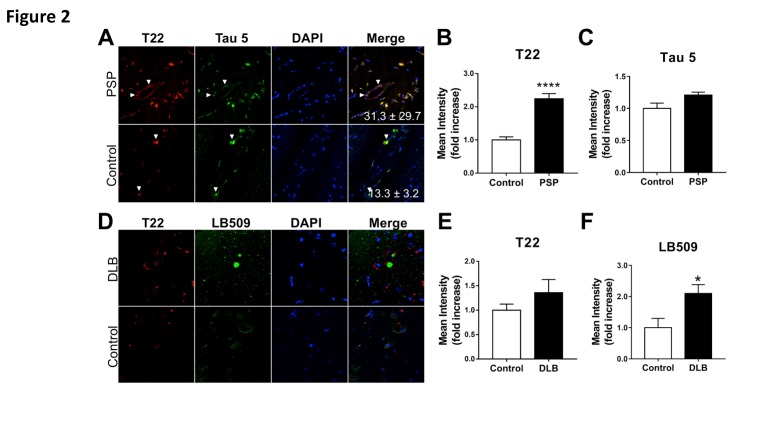
Increased deposition of tau oligomers in cerebrovasculature of patients with progressive supranuclear palsy (PSP) but not with dementia with Lewy bodies (DLB) **(A)** Representative images of pons sections from PSP patients and age-matched controls immunostained with antibodies specific for tau oligomers (T22, red) and total tau (Tau 5, green). Quantitative analyses of mean fluorescent intensity shows **(B)** increased levels of tau oligomers [****, t (18) = 7.38, *p*<0.0001] but not of total tau [**C**, t(7) = 1.67, *p* = 0.138] in cerebrovasculature of patients with PSP compared to age-matched controls. Examples of cerebrovascular oligomeric tau deposits are indicated with white arrows. **(D)** Representative images of brain sections from frontal cortex of DLB patients and age-matched controls immunostained with antibodies specific for tau oligomers (T22, red) and alpha-synuclein (LB509, green). **(E)** Quantitative analysis of mean fluorescence intensity did not reveal differences in oligomeric tau immunoreactivity in DLB patients compared to age-matched controls (t(9) = 1.289, *p* = 0.23). **(F)** Quantitative analysis of mean fluorescence intensity demonstrates an increase in alpha-synuclein abundance in brains of DLB patients compared to controls (*, t(9)=2.486, *p* = 0.035). For all studies, n=2 brains/group; 10-15 sections from each sample were analyzed for tau oligomers. All PSP and DLB samples were tested and were positive for tau oligomers.

## RESULTS

### Deposition of tau oligomers in AD brain vasculature

Bright field images of both AD (Fig. 1A-C) and control (Fig. 1D-F) cortical brain sections stained for T22 revealed tau oligomers present within small blood vessel walls in AD brains; these deposits are notably absent in age-matched controls. To further investigate these findings, we utilized confocal microscopy to obtain fluorescent images of both oligomeric and total tau protein. Our findings indicate increased colocalization of tau oligomers with Tau 5 in AD brains (Fig. 1G-I and J-L, with Mander’s coefficients shown in white) compared to age-matched controls (Fig. 1M-O). Furthermore, the mean intensity of T22 was increased nearly 100% per vascular element in AD brains (Fig. 1P) while the mean intensity of Tau 5 was increased by nearly 50% in the same subjects (Fig. 1Q) when compared to controls. These data indicate that tau oligomers accumulate in AD brain vasculature.

**Figure 3.**
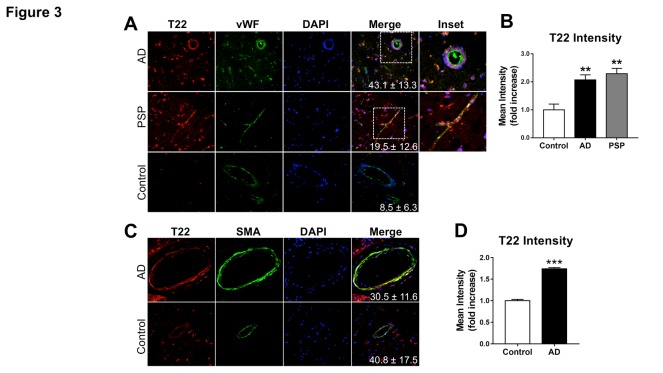
Association of tau oligomers with vascular endothelium and smooth muscle in Alzheimer’s disease brain Representative images of brain sections from AD patients and age-matched controls reacted with **(A)** antibodies specific for tau oligomers (T22, red) and the endothelial cell marker Von Willebrand Factor (vWF, green). **(B)** Quantitative analysis shows increased tau oligomer immuonoreactivity in the cerebrovasculature of AD and PSP patients compared to age-matched controls [Control-AD **, q=5.24 and *p*=0.0078, Control-PSP **, q=6.728 and p=0.0013, AD-PSP, q=1.03 and *p*=0.7517 as a result of Tukey’s *post hoc* test applied to a significant effect of group in ANOVA, F(2,12)=13.03, p=0.001]. **(C)** Representative images of brain sections from AD patients and age-matched controls reacted with antibodies specific for tau oligomers (T22, red) and the vascular smooth muscle cell marker smooth muscle actin (SMA, green). **(D)** Quantitative analysis demonstrates increased tau oligomer immuonoreactivity associated with smooth muscle actin-positive cells [***, t(4)=15.35, *p*=0.0001). For all studies, n=2 brains/group; 10-15 sections from each sample were analyzed for tau oligomers. All AD and PSP samples were tested and were positive for tau oligomers. Mean percent colocalization values ± SEM are shown as insets. Data in panels B and D were also included in the analyses of tau oligomer abundance in Figure 1P.

### Tau oligomers accumulate in cerebrovasculature of PSP and DLB patients

In order to determine whether vascular deposition of tau oligomers is common amongst tauopathies, we next determined tau oligomer deposition in the cerebrovasculature of PSP patients. Confocal images from the pons of PSP patients (Fig. 2A, upper panel) and age-matched control subjects (Fig. 2A, lower panel), were collected from sections immunostained using T22 and Tau 5 antibodies. Similar to our findings in AD subjects (Fig. 1), oligomeric tau immunoreactivity colocalized with Tau 5 immunoreactivity in vasculature of PSP brains and was largely absent in brains of age-matched control subjects. The mean intensity of oligomeric tau-specific immunoreactivity increased more than 100% in PSP subjects compared to age-matched controls (Fig. 2B), whereas a minimal and non-significant increase in total tau abundance was observed (Fig. 2C). These data indicate that, similar to our observations in AD brain (Fig. 1), tau oligomers preferentially accumulate in PSP cerebrovasculature.

We next determined localization and abundance of oligomeric tau and α-synuclein in sections from frontal cortex of patients with DLB using immunohistochemistry with an α-synuclein specific antibody (LB509) and T22. Our studies revealed deposition of tau oligomers in microvessel walls as well as in brain parenchyma of DLB brains (Fig. 2D, upper panel). Notably, Lewy body deposits were absent in microvasculature, but present as neuronal cytoplasmic deposits (arrow in Fig. 2D, upper panel) in the vicinity of blood vessels (Fig. 2D). Both oligomeric tau and α-synuclein immunoreactivity were absent in control subjects (Fig. 2D, lower panel). Although a trend to increased oligomeric tau immunoreactivity was observed in brain microvasculature of DLB patients, this difference was not significant (Fig. 2E). As expected, we observed a significant increase in α-synuclein immunofluorescence in DLB subjects (Figure 2F).

### Tau oligomers are associated with endothelial cell markers in AD and PSP

We next sought to determine whether oligomeric tau associates with specific brain vascular cell types in AD brain. Cortical sections from AD as well as pons sections from PSP and age matched control brains were immunostained with T22 and with antibodies specific for the endothelial cell marker von Willebrand Factor (vWF). Tau oligomers were found in association with brain vascular endothelial cells in AD and PSP (Fig. 3A) and were virtually absent in control brains. Immuno-fluorescence intensity of T22 associated with the brain vascular endothelial cell compartment, however, was significantly increased in AD and PSP brains (Fig. 3B). These data are consistent with the observed overall increases in cerebrovascular oligomeric tau as well as total tau in AD (Fig. 1) and PSP (Fig. 2A-C) brains, and suggest that tau oligomers are found in association with brain vascular endothelial cells in various neurological disease states.

Smooth muscle cells are a critical component of brain artery and arteriole walls that participate in the regulation of blood flow [27]. We next sought to determine whether in addition to targeting vascular endothelial cells, tau oligomers associate with vascular smooth muscle cells in AD. To this aim we used confocal imaging on sections from AD patients and age-matched controls immune-stained with T22 and with an antibody specific for smooth muscle actin (SMA), a marker of smooth muscle cells. Significant tau oligomer immunoreactivity was found in association with SMA immunoreactivity in arterioles in AD brain (Fig. 3C-D), suggesting that smooth muscle cells may also be a target of tau oligomer deposition. Together with our studies of Figure 1, these data demonstrate increased oligomeric tau in the cerebrovasculature of patients with AD compared to age-matched controls (Fig. 3D).

### Tau oligomers and Aβ partially colocalize in cerebrovasculature of the Tg2576 mouse model of AD.

**Figure 4.**
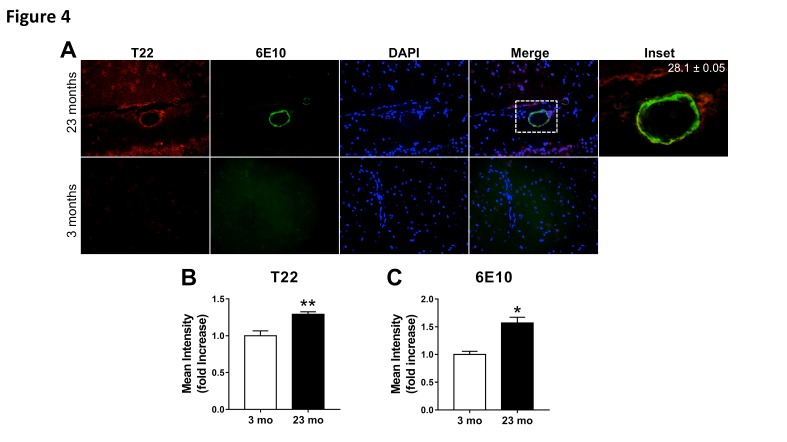
Age-dependent increase of oligomeric tau and fibrillar amyloid pathology in cerebrovasculature of Tg2576 mice **(A)** Representative images of brain sections from 23-month-old (top) and 3-month-old (bottom) transgenic Tg2576 mice reacted with antibodies specific for tau oligomers (T22, red) and amyloid-β (Aβ, 6E10, green). Mander’s colocalization coefficient suggests partial association of oligomeric tau and fibrillar Aβ in cerebrovasculature of 23-month-old mice. Because both T22 and Aβ immunoreactivity were absent in 3-month-old mice, a colocalization coefficient could not be computed for this group. Mean percent colocalization ± SEM are shown in the inset, which was merged without DAPI. **(B)** Quantitative analysis of T22-specific immunoreactivity shows increased cerebrovascular oligomeric tau deposition in 23 month-old compared to 3 month-old Tg2576 mice (**, t(19)=3.87, *p*=0.010). **(C)** Quantitative analysis of 6E10-specific immunoreactivity shows an age-dependent increase in cerebrovascular Aβ deposition in 23 month-old compared to 3 month-old mice (*, t(20)=2.62, p=0.016). n=3; 10-15 sections from each sample were analyzed for tau oligomers. All sections were positive for tau oligomers. All of the transgenic Tg2576 mice used were positive for both Abeta and tau oligomers.

A large body of evidence indicates that exposure to high levels of soluble forms of Aβ [28, 29] and fibrillar Aβ deposition [30] are deleterious to cerebrovascular function [4]. Because we showed that oligomeric tau accumulates in cerebrovascular endothelial and smooth muscle cells in various tauopathies including AD, we next sought to determine whether vascular Aβ and oligomeric tau may coexist in the cerebrovasculature. To this aim we used Tg2576 mice, which develop CAA-like cerebrovascular lesions starting at ~ 11-12 months of age that increase significantly with age, but are devoid of vascular fibrillar Aβ at earlier ages [26, 31]. Tg2576 mice are largely presymptomatic at 3 months of age, as synaptic loss [32], alterations in neuronal plasticity, and decline in cognitive function do not arise until 4-5 months of age [33]. Oligomeric tau and Aβ colocalization were measured in the cerebrovasculature of 3 and 23-month-old Tg2576 animals. We report abundant immunoreactivity for both tau oligomers and Aβ in cerebrovasculature of Tg2576 animals at 23 months of age (Fig. 4A, upper panel), in contrast with absent cerebrovascular tau oligomer and Aβ immunoreactivity in 3-month-old Tg2576 mice (Fig. 4A, lower panel). Notably, 28% of tau oligomers colocalize with Aβ within the cerebrovasculature of 23-month-old Tg2576 mice (Fig. 4A, inset). As expected, we demonstrated an age dependent increase in oligomeric tau immunofluorescence (Fig. 4B) and Aβ immunoreactivity (Fig. 4C) in 23-month-old Tg2576 mice compared to 3-month-old mice. It is conceivable that Aβ and oligomeric tau may functionally and potentially physically interact in AD cerebrovasculature, and may cooperatively act to compromise brain vascular function in AD.

## DISCUSSION

The major finding of the present study is that tau oligomers accumulate in cerebral microvasculature of human patients with AD and PSP, in association with vascular endothelial and smooth muscle cells. Cerebrovascular deposition of tau oligomers was also found in DLB patients, albeit to a lesser extent than in AD and PSP. Further, our studies show that tau oligomers accumulate in cerebral microvasculature of Tg2576 mice, a model that recapitulates CAA, at advanced stages of AD-like progression and vascular histopathology, and that they partially associate with cerebrovascular Aβ lesions.

These findings have important relevance for studies pertaining to the microvascular etiology of AD. Recent studies from our laboratory and others demonstrated tau oligomers constitute a distinct toxic species in AD [18, 20, 34, 35]. On the basis of previous *in vitro* findings showing that tau oligomers are cytotoxic at low nanomolar concentrations [36, 37] we predict that cerebral microvascular accumulation of tau oligomers impairs the function of endothelial cells and smooth muscle cells. Significant evidence exists that cerebral microvascular dysfunction contributes to the pathogenesis of AD [1-3]. Thus, future studies should determine how oligomeric tau impacts neurovascular coupling mechanisms, endothelial function and blood brain barrier integrity in AD and other tauopathies.

Cerebral microbleeds are small chronic brain hemorrhages [9-11] caused by structural weakening of the cerebral microvessels, which exacerbate brain damage and functional decline in AD patients. In theory, deposition of oligomeric tau in the cerebral microvessels may contribute to the pathogenesis of cerebral microbleeds by causing localized cell death and/or structural remodeling of the extracellular matrix. These possibilities should be tested in future studies.

Recently we demonstrated that specific removal of tau oligomers through passive immunotherapy using a tau oligomer-specific monoclonal antibody improves cognitive function without disrupting large NFTs in several tauopathy mouse models including the Tg2576, P301L, and Htau mice [38-41]. In light of our present findings further studies are warranted to determine whether immunotherapy using a tau oligomer-specific monoclonal antibody also removes tau oligomers from the cerebral microvessels and whether this improves/restores cerebral microvascular function. Initial evidence show that reducing tau levels rescues blood brain barrier integrity [42], suggesting that such experiments are worth undertaking.

The accumulation of Aβ in cerebrovasculature is common in AD and can occur in the absence of specific AD changes both sporadically as well as in association with specific familial mutations in the APP gene [43]. The potential for adjacent deposition of oligomeric tau in cerebral microvessels [44] raises the possibility that tau aggravates microvascular Aβ deposition and its consequences in AD.

In conclusion, our findings add to the growing evidence for multifaceted microvascular involvement in the pathogenesis of AD and other neurodegenerative diseases. Accumulation of tau oligomers represents a potential novel mechanism by which functional and structural integrity of the cerebral microvessels may be compromised in dementias.
